# Arterial Load and Norepinephrine Are Associated With the Response of the Cardiovascular System to Fluid Expansion

**DOI:** 10.3389/fphys.2021.707832

**Published:** 2021-08-04

**Authors:** Maxime Nguyen, Jihad Mallat, Julien Marc, Osama Abou-Arab, Bélaïd Bouhemad, Pierre-Grégoire Guinot

**Affiliations:** ^1^Department of Anesthesiology and Intensive Care, Centre Hospitalier Universitaire, Dijon, France; ^2^Lipness Team, INSERM Research Center LNC-UMR 1231 and LabExLipSTIC, University of Burgundy, Dijon, France; ^3^Department of Anaesthesiology and Intensive Care, Centre Hospitalier, Lens, France; ^4^Department of Anaesthesiology and Intensive Care, Centre Hospitalier Universitaire, Amiens, France

**Keywords:** arterial load, norepinephrine, fluid responsiveness, hemodynamic monitoring, sepsis and shock

## Abstract

**Background:**

Fluid responsiveness has been extensively studied by using the preload prism. The arterial load might be a factor modulating the fluid responsiveness. The norepinephrine (NE) administration increases the arterial load and modifies the vascular properties. The objective of the present study was to determine the relationship between fluid responsiveness, preload, arterial load, and NE use. We hypothesized that as a preload/arterial load, NE use may affect fluid responsiveness.

**Methods:**

The retrospective multicentered analysis of the pooled data from 446 patients monitored using the transpulmonary thermodilution before and after fluid expansion (FE) was performed. FE was standardized between intensive care units (ICUs). The comparison of patients with and without NE at the time of fluid infusion was performed. Stroke volume (SV) responsiveness was defined as an increase of more than 15% of SV following the FE. Pressure responsiveness was defined as an increase of more than 15% of mean arterial pressure (MAP) following the FE. Arterial elastance was used as a surrogate for the arterial load.

**Results:**

A total of 244 patients were treated with NE and 202 were not treated with NE. By using the univariate analysis, arterial elastance was correlated to SV variations with FE. However, the SV variations were not associated with NE administration (26 [15; 46]% vs. 23 [10; 37]%, *p* = 0.12). By using the multivariate analysis, high arterial load and NE administration were associated with fluid responsiveness. The association between arterial elastance and fluid responsiveness was less important in patients treated with NE. Arterial compliance increased in the absence of NE, but it did not change in patients treated with NE (6 [−8; 19]% vs. 0 [−13; 15]%, *p* = 0.03). The changes in total peripheral and arterial elastance were less important in patients treated with NE (−8 [−17; 1]% vs. −11 [−20; 0]%, *p* < 0.05 and −10 [−19; 0]% vs. −16 [−24; 0]%, *p* = 0.01).

**Conclusion:**

The arterial load and NE administration were associated with fluid responsiveness. A high arterial load was associated with fluid responsiveness. In patients treated with NE, this association was lower, and the changes of arterial load following FE seemed to be driven mainly by its resistive component.

## Introduction

For several years, a number of literature has been published on fluid expansion (FE) during resuscitation of acute circulatory failure and perioperative hemodynamic optimization. The cardiovascular effects of FE are most often evaluated through the prism of pressure/outflow models (e.g., Guyton and Frank–Starling). Briefly, according to Frank–Starling theory, a fluid-responsive patient is a patient whose cardiac output (CO) increases with FE. According to the venous return curves as suggested by Guyton, FE increases the CO by increasing the venous return gradient (VRG) [and decreasing the resistance to venous return (RVR)]. By plotting together the venous return curves and the Frank–Starling curves, the physicians were able to describe the steady-state operating point defining the CO and right atrial pressure for a physiological condition (Guyton, 1955).

As cardiovascular homeostasis is a complex process, analyzing the cardiovascular effect of FE only through its effect on preload may imperfectly describe the dynamics of the interaction between the heart, the venous return, and the vascular system. In fact, it has been demonstrated that FE could increase the CO without increasing the preload (Kumar et al., 2004) and that an increase in the pressure of the right venous may not be associated with CO increase (Fleming and Bloom, 1957). Several mechanisms might explain those observations. First, it has been demonstrated that modification in the arterial load (i.e., the opposition of the vascular system to cardiac ejection) might modulate the CO in order to keep the tissular perfusion constant (Guyton, 1981) and that FE may change the arterial load in critically ill patients (Monge García et al., 2015; Huette et al., 2020). Second, norepinephrine (NE) is also likely to interact with fluid responsiveness. In fact, by increasing the cardiac preload, NE might reduce the preload dependency (Monnet et al., 2011), and vasopressor administration might decrease the effect of FE by altering the vascular characteristics (Vane et al., 2004). Thus, NE might affect both preload and arterial load, and its administration could be associated with the changes in non-preload stroke volume (SV) (Guinot et al., 2018). Altogether, the arterial load and NE administration are likely to be key players in fluid responsiveness. To date, no study has evaluated these associations.

The objective of the present study was to determine the relationship between fluid responsiveness, arterial load, and NE use. In addition, we aimed to describe the effect of NE on the changes induced by FE in preload and arterial load. We hypothesized that preload, baseline arterial load, and NE administration may impair fluid responsiveness.

## Materials and Methods

### Patients

The authors pooled the data obtained from three databases (i.e., CHU d’Amiens, CHU de Dijon, and CH de Lens). The patients received oral information prior to its inclusion into those databases. The study was performed in accordance with the ethical standards of the 1964 Declaration of Helsinki. As the study was observational and retrospective, according to French law, we did not need written consent (Loi Jardé 5 mars, 2012). We specifically consulted the institutional review board (IRB) (i.e., ethical committee) of the French Society of Anaesthesia and Intensive Care Medicine for the present retrospective analysis, which confirmed that this study did not present any ethical concern and that no written consent was needed (IRB 00010254-2019 – 180).

The main inclusion criteria were as follows: age 18 years or above, controlled positive ventilation, and a clinical decision to perform FE for volume expansion. The non-inclusion criteria were arrhythmia, aortic regurgitation, and known dysfunction of the right heart. Sepsis was defined according to the International Sepsis Definitions Conference (Levy et al., 2003). The present manuscript was drafted in compliance with the Strengthening the Reporting of Observational Studies in Epidemiology checklist for cohort studies.

### Study Procedures

All patients were monitored with arterial catheterization, central venous pressure (CVP), and transpulmonary thermodilution (PICCO, Getinge, France, Orléans). FE was standardized between intensive care units (ICUs), and it always consisted of an FE of 500 ml of crystalloid over 10 min. A transpulmonary thermodilution was performed immediately after the FE to measure the CO. The following clinical parameters were recorded: demographic parameters, ventilation parameters, and primary diagnosis. After an equilibration period, parameters such as heart rate (HR), systolic arterial pressure, mean arterial pressure (MAP), diastolic arterial pressure, CVP, SV, CO, and pulse pressure (PP) were measured. Immediately after FE, a second set of measurements was made. All patients underwent mechanical ventilation in a volume-controlled mode. Ventilator settings (e.g., oxygen inspired fraction, tidal volume, respiratory rate, and end positive pressure) were not modified during the study period.

### Hemodynamic Parameters, Venous Return, and Arterial Elastance

Sunagawa et al. (1983, 1985) demonstrated that the arterial load could be characterized in the time domain as arterial elastance (*E*_*A*_). *E*_*A*_ was estimated by using the equation *E*_*A*_ = TVR/*T* + 0.42/*C*_*A*_ − 0.04, where *T* is the cycle length (Chemla et al., 2003). The assessment of arterial load was based on a two-element Windkessel model that comprises total peripheral resistance (TPR) and arterial compliance (*C*_*A*_) (Chemla et al., 2003). The TPR (mmHg/ml/min) was calculated as MAP − CVP/CO. The *C*_*A*_ (ml/mmHg) was calculated as SV/arterial PP (Chemla et al., 2003). The mean systemic filling pressure (MSFP) was calculated by using a mathematical model based on anthropometric variables (e.g., age, height, and weight) and direct measures of CO, CVP, and MAP (Parkin and Leaning, 2008). The VRG (mmHg) was calculated as: MSFP − CVP. RVR (mmHg/ml/min) was calculated as MSFP − CVP/CO. The overall pumping efficiency of the heart was estimated as Eh = MSFP − CVP/MSFP (Parkin and Leaning, 2008).

### Statistical Analysis

The data are expressed as number, proportion (in percent), or median [interquartile range]. The qualitative data were compared using the chi-squared test or Fisher’s exact test. For the quantitative data, normality was assessed using the Shapiro–Wilk test. Accordingly, the data were compared by using Student’s *t*-test or Kruskal–Wallis sum rank test as appropriate. The paired data were compared with the paired Student’s *t*-test or the Wilcoxon signed rank test. The changes in the hemodynamic variables were expressed as the percentage from the baseline value. The CVP variations were expressed as (CVP after − CVP before)/[(CVP after + CVP before)/2]. The patients were classified into fluid responders and non-responders as a function of the effect of FE on SV. Fluid response was defined as an increase of more than 15% of the SV after FE (Guinot and Lorne, 2015). A focused principal component analysis (FPCA) was carried out to explore the relationship between the SV variations induced by FE and the hemodynamic status at the baseline. The FPCA allows representing both the correlation between the variable of interest (i.e., SV variations) and multiple baseline hemodynamic variable and the correlation of those variables between each other. The variable of interest (i.e., SV variations) is represented in the center of the graph. The strength of the correlation between baseline hemodynamic values and SV variation is represented by the distance to the center of the circle: the higher the strength, the closer to the center of the circle. The sign of the correlation is represented by the color: blue for positive correlation and red for negative correlation. The hemodynamic baseline that is correlated together appears close to each other. The missing values were imputed by ACP only for the representation of the focused ACP. A logistic regression model was carried out to assess the relationship between the fluid response and arterial load, venous return indexes, NE, and the main confounders identified in the literature and by the univariate analysis. The variable selection was stepwise, based on both physiology and Akaike information criterion. The collinearity was addressed by using regression coefficient. Goodness of fit was assessed by the Hosmer–Lemeshow method. The threshold for statistical significance was set to *p* < 0.05. The RStudio software (Version 1.1.447 –© 2009–2018 RStudio, Inc.) was used for all statistical analyses.

## Results

### Baseline Characteristics

**FIGURE 1:**
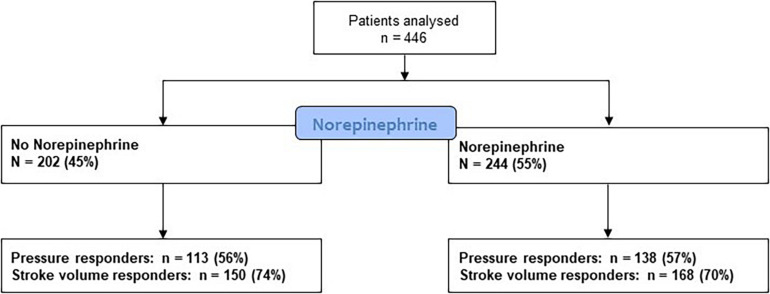
Flowchart of the study.

A total of 446 patients were included in the present study (Figure 1). The median age was 69 [60; 77] years, and 58% of the patients were men. The median body mass index was 27 [24; 30], and 30% of the patients had ongoing sepsis and 244 (55%) patients were treated with NE with a median dose of 0.20 [0.07; 0.72] μg/kg/min. Furthermore, 138 (71%) of patients were SV responders to FE, and 251 (56%) were pressure responders.

At the baseline, the SV was not significantly different between patients receiving NE and patients without NE (51 [39; 63] ml vs. 49 [34; 64] ml, *p* = 0.49). The HR was higher in the NE group (83 [72; 107] bpm vs. 74 [64; 88] bpm, *p* < 0.01) (Table 1). The patients treated with NE also had higher arterial blood pressure, CVP, MSFP, VRG, and lower RVR and overall pumping efficiency. The PP was not different between groups. The *C*_*A*_ and *E*_*A*_ did not significantly differ between the two groups, but the TPR was higher in patients without NE (Table 1).

**TABLE 1:** Hemodynamic parameters, before and after fluid expansion, depending on norepinephrine administration.

Variable	No norepinephrine *N* = 202	Norepinephrine *N* = 244	*p*-value
**Stroke volume (ml)**			
Before	51 [39; 63]	49 [34; 64]	0.49
After	63 [50; 79]	63 [45; 81]	0.39
**Heart rate (bpm)**			
Before	74 [64; 88]	83 [72; 107]	< 0.01
After	72 [62; 85]	83 [70; 102]	< 0.01
**Mean arterial pressure (mmHg)**			
Before	67 [61; 75]	71 [60; 79]	< 0.01
After	76 [68; 86]	81 [73; 88]	< 0.01
**Pulse pressure (mmHg)**			
Before	46 [39; 57]	49 [37; 60]	0.30
After	57 [47; 69]	61 [47; 74]	0.16
**Central venous pressure (mmHg)**			
Before	6 [4; 8]	9 [6; 12]	< 0.01
After	9 [6; 11]	11 [9; 15]	< 0.01
**Mean systemic filling pressure (mmHg)**			
Before	11 [9; 14]	15 [12; 19]	< 0.01
After	15 [12; 18]	18 [15; 22]	< 0.01
**Venous return gradient (mmHg)**			
Before	6 [5; 6]	6 [5; 7]	0.02
After	7 [6; 7]	7 [6; 8]	< 0.01
**Resistance to venous return (mmHg/min/l)**			
Before	1.48 [1.32; 1.84]	1.44 [1.24; 1.77]	< 0.01
After	1.40 [1.26; 1.74]	1.38 [1.18; 1.60]	0.02
**Overall pumping efficiency**			
Before	0.48 [0.40; 0.62]	0.40 [0.33; 0.51]	< 0.01
After	0.45 [0.38; 0.52]	0.38 [0.32; 0.48]	< 0.01
**Arterial elastance (mmHg/ml)**			
Before	2.00 [1.52; 2.80]	2.15 [1.50; 3.10]	0.55
After	1.60 [1.20; 2.10]	1.80 [1.40; 2.40]	0.02
**Arterial compliance (ml/mmHg)**			
Before	1.10 [0.80; 1.40]	1.00 [0.78; 1.30]	0.20
After	1.10 [0.82; 1.50]	1.00 [0.80; 1.40]	0.04
**Total peripheral resistance (mmHg/min/ml)**			
Before	17.0 [13.0; 22.0]	15.0 [11.0; 20.2]	< 0.01
After	15.0 [11.0; 19.0]	14.0 [10.0; 18.0]	0.01

**p*-value refer to between groups comparisons. Student *t*-test or the Kruskal–Wallis test was used for between group comparison.*

### Parameters After FE

After FE, SV was not significantly different between groups. The HR and MAP remained higher in patients with NE. The PP did not differ between groups. The CVP, MSFP, VRG, and *E*_*A*_ were higher in patients treated with NE. On the contrary, RVR, overall pumping efficiency, *C*_*A*_, and TPR were higher in patients without NE.

### Percent Changes of Parameters Caused by FE

The hemodynamic changes following FE differed between patients with/without NE (Table 2). The changes in SV, HR, MAP, and PP were not significantly different between groups (*p* > 0.05). Regarding the changes following FE, in patients without NE: CVP and MSFP were higher (33 [18; 58]% vs. 24 [9; 40]%, *p* < 0.01 and 30 [18; 44]% vs. 21 [11; 37]%, *p* < 0.01, respectively), but there was no significant difference in increase of VRG (17 [13; 35]% vs. 17 [0; 33]%, *p* = 0.63). The RVR and overall pumping efficiency decreased further.

**TABLE 2:** Hemodynamic variations depending on the presence of norepinephrine.

Variations with fluid expansion (%)	No norepinephrine *N* = 202	Norepinephrine *N* = 244	*p*-value
Stroke volume	26 [15; 46]	23 [10; 37]	0.12
Heart rate	−3 [−7; 0]	−2 [−7; 1]	0.23
Mean arterial pressure	12 [4; 20]	13 [4; 22]	0.69
Pulse pressure	20 [9; 37]	22 [8; 43]	0.66
Central venous pressure	33 [18; 57]	24 [9; 40]	< 0.01
Mean systemic filling pressure	30 [18; 44]	21 [11; 37]	< 0.01
Venous return gradient	17 [13; 25]	17 [0; 33]	0.63
Resistance to venous return	−5 [−9; −1]	−3 [−7; 1]	0.02
Overall pumping efficiency	−10 [−21; 0]	−6 [−14; 6]	< 0.01
Arterial compliance	6 [−8; 19]	0 [−13; 15]	0.03
Arterial elastance	−16 [−−24; 0]	−10 [−19; 0]	0.01
Total peripheral resistance	−11 [−20; 0]	−8 [−17; 1]	< 0.05

*Results are expressed as percentage from baseline value. *p*-value refer to between groups comparison. Student’s *t*-test or Kruskal–Wallis sum rank test were used.*

The *C*_*A*_ increased in the absence of NE, but it did not change in patients treated with NE (6 [−8; 19]% vs. 0 [−13; 15]%, *p* = 0.03) (Table 2). The changes in total peripheral and *E*_*A*_ were less important in patients with NE (−8 [−17; 1]% vs. −11 [−20; 0]%, *p* < 0.05 and −10 [−19; 0]% vs. −16 [−24; 0]%, *p* = 0.01).

### Focused Principal Component Analysis

**FIGURE 2:**
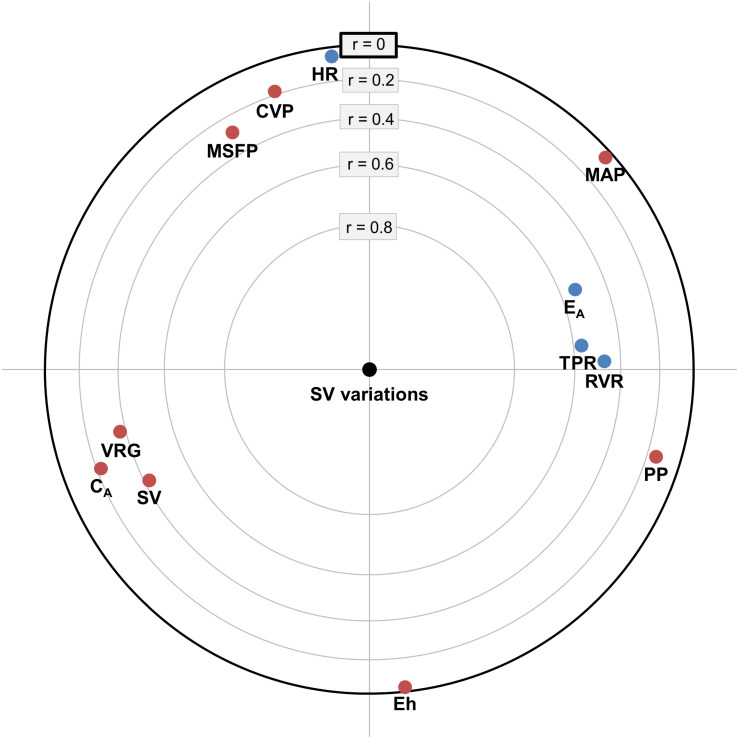
Focused principal component analysis representing stroke volume (SV) variations depending on the hemodynamic variable at baseline. The center of the circle is the variable of interest (i.e., SV variations). Baseline hemodynamic variables highly correlated with SV variations appear close to the center of the circle, positive correlations being represented in blue and negative correlations in red. Hemodynamic variables that are correlated together appear close to each other in the two-dimensional space. *C*_*A*_, arterial compliance; CVP, central venous pressure; *E*_*A*_, arterial elastance; Eh, overall pumping efficiency; HR, heart rate; MAP, mean arterial pressure; MSFP, mean systemic filling pressure; NE, norepinephrine; PP, pulse pressure; RVR, resistance to venous return; SV, stroke volume; TPR, total peripheral resistance; VRG, venous return gradient.

A FPCA was carried out in order to explore the relationship between SV variations and baseline hemodynamic variables. The FPCA shows that the variables (e.g., *E*_*A*_ and TPR) related to the arterial load were correlated with the RVR. Those variables appeared moderately correlated to SV variations (Figure 2). On the contrary, *C*_*A*_, baseline SV, VRG, MSFP, and CVP were negatively correlated with SV variations.

### Multivariate Analysis

By using the multivariate analysis (Table 3), fluid responsiveness was associated with the high baseline arterial load (i.e., *E*_*A*_) and NE use. The association between fluid responsiveness and arterial load was lower in the presence of NE (i.e., negative interaction). The low CVP and MAP were associated with fluid responsiveness. The patients with sepsis were less fluid responders.

**TABLE 3:** Multivariate logistic regression analysis analyzing factor associated with stroke volume response.

Variable	ORa (confidence interval)	*p*-value
Arterial elastance	2.4 [1.4; 4.3]	< *0.01*
Norepinephrine	10.9 [3.2; 39.3]	< *0.01*
Interaction *E*_*A*_ and norepinephrine	0.50 [0.28; 0.82]	< *0.01*
Central venous pressure	0.94 [0.88; 1.00]	*0*.*04*
Venous return gradient	0.85 [0.69; 1.05]	0.13
Mean arterial pressure	0.97 [0.94; 1.00]	*0*.*04*
Sepsis	0.36 [0.14; 0.88]	*0*.*03*
Medical admission	0.73 [0.31; 1.77]	0.47
Heart rate	1.0 [0.98; 1.01]	0.60

## Discussion

The main result of this study is that the high arterial load and NE administration were associated with fluid responsiveness. Since NE changes the vascular properties, it decreased the association between fluid responsiveness and arterial load. In the patients treated with NE, the changes of *E*_*A*_ seemed to be driven mainly by its resistive component (i.e., TPR), whereas the pulsatile component (i.e., *C*_*A*_) did not change.

To date, few studies have focused on fluid responsiveness by analyzing the preload and arterial load components and their interaction with the changes in cardiac blood flow all together. In our results, a higher arterial load was associated with fluid responsiveness. To the best of our knowledge, this relationship has never been previously documented. This result may be explained by using several factors. First, the fluid-responsive patients had a higher arterial load reflecting higher TPR and/or lower *C*_*A*_. This hemodynamic status may traduce the activation of physiological mechanisms to maintain the tissue perfusion in case of low CO. Second, ventricular elastance has been demonstrated to be unchanged by FE (Chang et al., 1997; Huette et al., 2020). Thus, any change of the arterial load was likely to impact ventriculo-arterial coupling (Sunagawa et al., 1983; Guinot et al., 2015). At baseline, a high *E*_*A*_ may result in ventriculo-arterial uncoupling and may impair cardiac ejection. Since FE decreased the *E*_*A*_, it is likely that FE improved ventriculo-arterial coupling, thus SV (Huette et al., 2020). In non-fluid responders, a low SV response to FE may be explained by lower *E*_*A*_ and better ventriculo-arterial coupling before FE. Altogether, our results support that monitoring arterial load and ventriculo-arterial coupling might bring further explanation on the effect of treatment on hemodynamic status, thus helping tailoring therapeutics (Guarracino et al., 2013, 2019; Guinot et al., 2018).

Although the arterial load is known to increase with NE administration (Nguyen et al., 2020), we observed a similar baseline *E*_*A*_ in the patients with NE and without NE. FE decreased *E*_*A*_, but this mechanism was dependent on NE use. When considering the patients free of NE, a decrease in *E*_*A*_ might be caused by several interlinked mechanisms affecting its resistive component (i.e., TPR) and its pulsatile component (i.e., *C*_*A*_). The sympathetic nervous system plays a key role in regulating blood flow and pressure (Wehrwein and Joyner, 2013) and the baroreflex maintains adequate blood pressure by modulating both the vascular properties [i.e., arterial resistance (thus *E*_*A*_) and the stressed blood volume] and CO (Sakamoto et al., 2015). Furthermore, FE initiates nitric oxide–dependent vasodilatation (Calver et al., 1992) (suggesting a drop in arterial load), and blood flow might modulate the diameter (thus compliance) of the large blood vessels (Snow et al., 1994). In this cohort study, it seemed that NE interfered with those mechanisms by fixing the pulsatile component of the arterial load. Indeed, the vascular response to FE was different in patients treated with NE: *E*_*A*_ and TPR decrease was of lesser magnitude, and *C*_*A*_ did not change.

Finally, our results might explain the conflicting results on the ability of dynamic *E*_*A*_ to predict the pressure response to FE (Monge Garcia et al., 2011; Lanchon et al., 2017). The dynamic arterial elastance (Ea_*dyn*_) is a measure of vascular load depending on *C*_*A*_ and vascular resistance (Pinsky, 2012; Bar et al., 2018a,b; Nguyen et al., 2021). If NE fixes *C*_*A*_, Ea_*dyn*_ may better reflect vascular resistance in this context. On the contrary, Ea_*dyn*_ may vary in a more complex manner without NE, decreasing the ability to predict the changes in blood pressure following volume expansion. Regarding Ea_*dyn*_ accuracy, operative patients are usually less exposed to vasopressors, and as the positive studies were mostly conducted in the ICU, the main negative studies were performed in the operating theatre (Monge Garcia et al., 2011; Guinot et al., 2015; Lanchon et al., 2017).

Some limitations can be discussed. The present study is a retrospective analysis of databases and only association but not causality could be inferred. The arterial load was estimated from *E*_*A*_ by femoral catheterization; however, non-invasive evaluation, *E*_*A*_, was validated against the gold standard method (Kelly et al., 1992). We did not measure ventricular elastance but by definition, and as demonstrated in previous studies, FE did not change ventricular elastance (Huette et al., 2020), and the main mechanism is an improvement of *E*_*A*_. The response to FE was evaluated based on SV changes because several studies have demonstrated that CO or blood pressure is not sufficiently sensitive and specific to track the effect of FE on blood flow (Lakhal et al., 2013; Guinot et al., 2014). Due to the power of the statistical analysis, some differences appeared statistically significant although clinically irrelevant, especially when the signed rank test was carried out.

In conclusion, the present results suggest that the arterial load and NE are determinants of fluid responsiveness. A higher *E*_*A*_ was associated with fluid responsiveness. The ongoing NE infusion was also associated with fluid responsiveness. NE impaired the association between fluid responsiveness and *E*_*A*_. In patients treated with NE, the changes of arterial load following FE seemed to be driven mainly by its resistive component.

## Data Availability Statement

The original contributions presented in the study are included in the article/supplementary material, further inquiries can be directed to the corresponding author.

## Ethics Statement

The studies involving human participants were reviewed and approved by the French Society of Anaesthesia and Intensive Care Medicine. Written informed consent for participation was not required for this study in accordance with the national legislation and the institutional requirements.

## Author Contributions

JMi, JMu, OA-A, and P-GG collected the data. JMi, P-GG, and MN analyzed the data. P-GG, BB, and MN prepared the manuscript. All authors contributed to the article and approved the submitted version.

## Conflict of Interest

The authors declare that the research was conducted in the absence of any commercial or financial relationships that could be construed as a potential conflict of interest.

## Publisher’s Note

All claims expressed in this article are solely those of the authors and do not necessarily represent those of their affiliated organizations, or those of the publisher, the editors and the reviewers. Any product that may be evaluated in this article, or claim that may be made by its manufacturer, is not guaranteed or endorsed by the publisher.

## Abbreviations

CO: cardiac output
*E*_*A*_: arterial elastance
Eh: overall pumping efficiency
FE: fluid expansion
HR: heart rate
ICU: intensive care unit
MAP: mean arterial pressure
MSFP: mean systemic filling pressure
NE: norepinephrine
PP: pulse pressure
RVR: resistance to venous return
SV: stroke volume
TPR: total peripheral resistance
VRG: venous return gradient.
